# Scalable High Refractive Index polystyrene-sulfur nanocomposites via in situ inverse vulcanization

**DOI:** 10.1038/s41598-020-71227-z

**Published:** 2020-09-10

**Authors:** Vijay S. Wadi, Kishore K. Jena, Kevin Halique, Brigita Rožič, Luka Cmok, Vasileios Tzitzios, Saeed M. Alhassan

**Affiliations:** 1 Department of Chemical Engineering, Khalifa University of Science and Technology, PO Box 127788, Abu Dhabi, UAE; 2 Institut “Jožef Stefan”, P.O. Box 3000, 1001 Ljubljana, Slovenia; 3 NCSR “Demokritos” Institute of Nanoscience and Nanotechnology, 15310 Athens, Greece

**Keywords:** Engineering, Nanoscience and technology, Optics and photonics

## Abstract

In this work, we demostrate the preparation of low cost High Refractive Index polystyrene-sulfur nanocomposites in one step by combining inverse vulcanization and melt extrusion method. Poly(sulfur-1,3-diisopropenylbenzene) (PS-SD) copolymer nanoparticles (5 to 10 wt%) were generated in the polystyrene matrix via in situ inverse vulcanization reaction during extrusion process. Formation of SD copolymer was confirmed by FTIR and Raman spectroscopy. SEM and TEM further confirms the presence of homogeneously dispersed SD nanoparticles in the size range of 5 nm. Thermal and mechanical properties of these nanocomposites are comparable with the pristine polystyrene. The transparent nanocomposites exhibits High Refractive Index n = 1.673 at 402.9 nm and Abbe’y number ~ 30 at 10 wt% of sulfur loading. The nanocomposites can be easily processed into mold, films and thin films by melt processing as well as solution casting techniques. Moreover, this one step preparation method is scalable and can be extend to the other polymers.

## Introduction

In recent years, optical materials with High Refractive Index (RI) have received much attention because of their wide variety of application in areas such as ophthalmic lenses, filters, optical adhesives, high reflective and antireflection coatings as well as advanced optoelectronic fabrications^1^. Inorganic materials usually possesses a high RI (n = 2.0–5.0)^2^, however, their lower flexibility and higher densities (> 2.5 g cm^3^) limits their applications^3^. On the other hand polymeric materials overcome these issues and attracted many researchers due to the advantages of low weight, excellent impact resistance, easy processability and low cost compared with inorganic materials^4^. Unfortunately, RI for majority of polymers lay below n = 1.3–1.6^5^. According to the Lorentz–Lorenz equation, refractive index of the polymers can be enhanced considerably by introducing a substituent with high molar refraction, low molar volume, high aromatic contents, or high density^6,7^. Thus, several methods were employed to achieve High Refractive Index (HRI) by introducing range of functional moieties such as aromatic rings, heteroaromatic rings, heavy atom halogen (Cl, Br, and I) and sulfur in the polymer; however, these methods involves extensive monomer and polymer synthesis procedure^8^. In another approach inorganic or metal nanoparticles like (ZrO_2_)^9,10^, (TiO_2_)^11–13^, ZnO^14^ etc., were added into a polymer matrix to produce polymer composites with HRI. It has been well established that sulfur atoms are effective in increasing refractive indices because of their high atomic refraction, hence the sulfur containing nanoparticles like FeS^15^, ZnS^16,17^ and PbS^18^ were also used as nano-fillers to increase the RI. However, dispersion of inorganic nanoparticles in the polymer matrix is highly challenging, moreover increasing concentration increases their agglomeration tendency which could significantly effect the transparency of the material and could limits their applications. Thus, polymer that contain sulfur in back bone including epoxy^19^, polyurethane^20^, polymethacrylate^21,22^, poly(thiocarbonate)s^23,24^, poly(thioether sulfone)^24^ and polyimide (PI)^25^ have been explored extensively for their HRI properties.

Direct utilization of elemental sulfur in producing HRI polymers are limited due to the low solubility and compatibility of sulfur in most organic solvents and chemicals^26^. Recently, Theato et al., reported the use of elemental sulfur in the preparation of polythioamide by Willgerodt–Kindler reaction with High Refractive Index of n = 1.87 at λ = 589.3 nm^27^. In another study Zhang et al., prepared polystyrene-sulfur composites with high Abbe’s number (27.3–30.4) and refractive index of n = 1.6220, in this method elemental sulfur was partially reacted with styrene and mixed with polystyrene in solution to improve the compatibility and dispersibility of the sulfur; which resulted in improving RI of the polymer^28^.

Recently, Pyun et al. prepared sulfur rich copolymers via inverse vulcanization technique, in this method linear sulfur chains were stabilized between organic molecules by forming highly cross-linked structure. The presence of these S–S bonds and the cross-linked structure imparted HRI n > 1.7 to the copolymers, moreover, S–S bonds are largely inactive in IR region and hence enabled to take high quality images in the near (1.5 μm) and mid‐IR (3–5 μm) regions^29,30^. Further enhancement in RI was achieved by adding inorganic selenium in the copolymer, the addition of selenium into the inverse vulcanized sulfur copolymers significantly improved the RI (n > 2) and showed excellent IR transparence^31^. More recently, Kleine, et al., developed chalcogenide hybrid inorganic/organic polymers with enhanced long-wave infrared (LWIR) spectrum (7–14 µm), this low organic content terpolymers showed superior IR transparence and demonstrated the ability to take highly resolved thermal images in near or complete dark environment^32,33^. Similar chemistry of introducing S–S bond in the copolymer to improve the refractive index was later adopted by many researchers are prepared various HRI copolymers^34–38^.

Taking advantage of these S–S bonds, herein, we report on the preparation of scalable polystyrene sulfur composites by one step melt extrusion technique with improved RI. In this method, cross-linked sulfur copolymer nanoparticles were generated inside the polystyrene matrix by in situ reaction of sulfur and DIB during extrusion process. The amount of sulfur in the composite was optimized to obtain transparent materials with High Refractive Index (RI) as well as Abbe’s number. In situ cross-linking reaction was confirmed by FTIR and Raman spectroscopy. The thermal properties were studied by DSC and TGA. Surface morphology and optical properties were estimated by SEM, TEM and UV–Vis spectroscopy respectively. Mechanical and thermomechanical properties of the composites were also studied and compared with pure polystyrene. The main advantage of the prepared composites is that these composites can be easily scalable and processable into films, molds and thin films by melt processing as well as solution casting method without sacrificing the mechanical properties.

## Result and discussion

**Figure 1:**
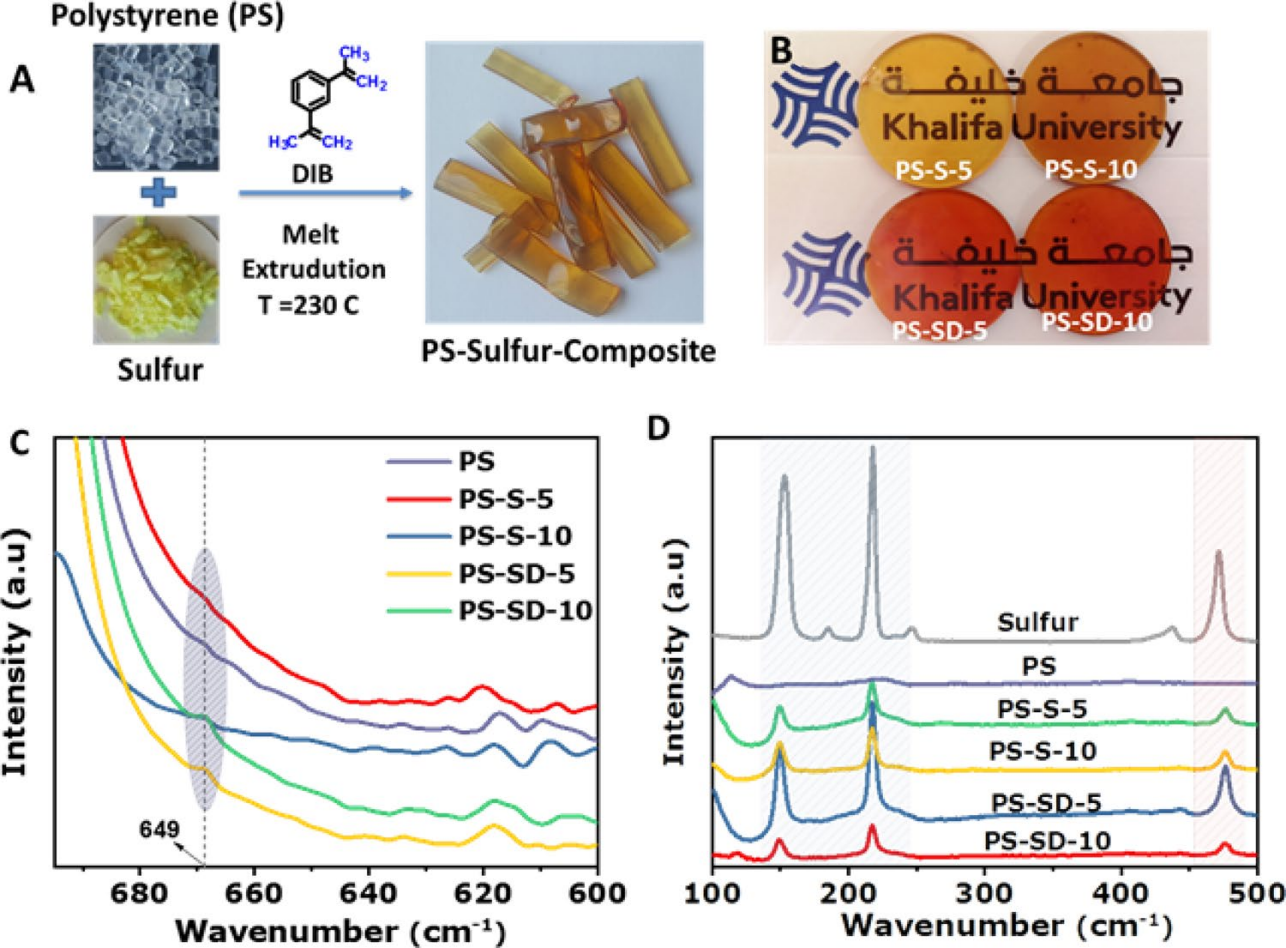
(**A**) In situ inverse vulcanization of sulfur and DIB in polystyrene matrix, (**B**) digital image of the processed circular disc of PS-S and PS-SD composites (**C**) FTIR and (**D**) Raman spectra of PS, PS-S and PS-SD composites with different sulfur loading, respectively.

Incorporation of elemental sulfur into polymers during synthesis is highly challenging due to its low solubility and compatibility with majority of organic chemicals. Further, polysulfur produced from heat treatment are highly brittle and unstable at room temperature; which also make it undesirable for use during polymer processing. Thus, in this method polysulfur chains are stabilized by reacting elemental sulfur with DIB cross-linker inside the polystyrene matrix during extrusion via in situ inverse vulcanization. A schematic representation for the preparation of transparent polystyrene-sulfur composites is shown in Fig. 1A.

Initially, pure polystyrene melted at 230 °C in an extruder, to this known amount of sulfur; DIB were added in stepwise and allowed to mix for 30 min. Under extrusion condition sulfur undergo ring opening polymerization to form a viscous polysulfur which later reacts with DIB to yield cross-linked SD copolymers. These viscous melts effectively mix with PS melts and diffuse into the matrix due to the applied shear resulting in a homogeneously dispersed transparent composites (see Fig. 1A). Colorless polystyrene changed to orange red color confirming the successful cross-linking reaction (see Fig. 1B bottom row). Similar color change was also observed when pure sulfur directly reacted with DIB (see Fig. S1). These cross-links provide structural stability to the polysulfur chains whereas DIB improves the compatibility with PS matrix. For comparison, composites were also prepared without the cross-linkers resulting in transparent yellow colored polystyrene-sulfur (PS-S) composites (see Fig. 1B top row). Optically transparent materials were obtained with up to 10 wt% of sulfur loading, excess addition resulted in agglomeration of particles and reduced transparency (see Fig. S2). Therefore, the composites with 5 and 10 wt% of sulfur loading were only prepared and used for further study.

The main advantage of these composites is their ability to scale up and processability into variety of different objects by melt and solution processing technique. Molded objects and films prepared by injection molding and hot-press methods are displayed in Fig. S3 are transparent and uniformly colored like extrudate indicate the stability and unchanged morphology of the composites. Thus, the nanocomposites are feasible to process into desire shape and size without altering the transparency. The composites also exhibits high solubility in most of organic solvent similar to polystyrene and form a clear and stable solution (see image Fig. S4); hence, enable to prepare ultra-thin films on glass slides by solution casting or spin coating method. Thus, the one step preparation and easy processability of polystyrene-sulfur composites have greater advantage over inorganic polymer composites.

In situ cross-linking reaction of SD copolymers was verified by carrying out Fourier-transform infrared spectroscopy (FT-IR) analysis (Fig. 1C). In the FT-IR spectrum of PS-SD samples the appearance of new stretching bands at 1,012 and 645 cm^−1^ corresponding to C–S bonding indicating successful chemical reaction between sulfur and DIB^39^. Intensities of these peaks are relatively weak due to the higher peak intensities of polystyrene in the same IR region. Moreover, absence of characteristic C=CH_2_ peak located at 900 cm^−1^ clearly indicates the complete consumption of DIB during cross-linking reaction. Interestingly, polystyrene peaks in the spectra’s are unchanged suggests the structural stability and non-reactivity of PS against sulfur under extrusion condition (see Fig. S6). This was also supported by solid state ^1^H-NMR spectra (see Fig. S7). The nature of carbon–sulfur bonding within the polymeric matrix of PS-SD was further elucidated by Raman spectroscopy analysis (see Fig. 1D). PS-SD showed S–S bonding peaks at 152, 220, and 474 cm^−1^ along with the ν (C–S) peak at 182 cm^−1^, which indicates successful chemical reaction of sulfur and DIB.

**Figure 2:**
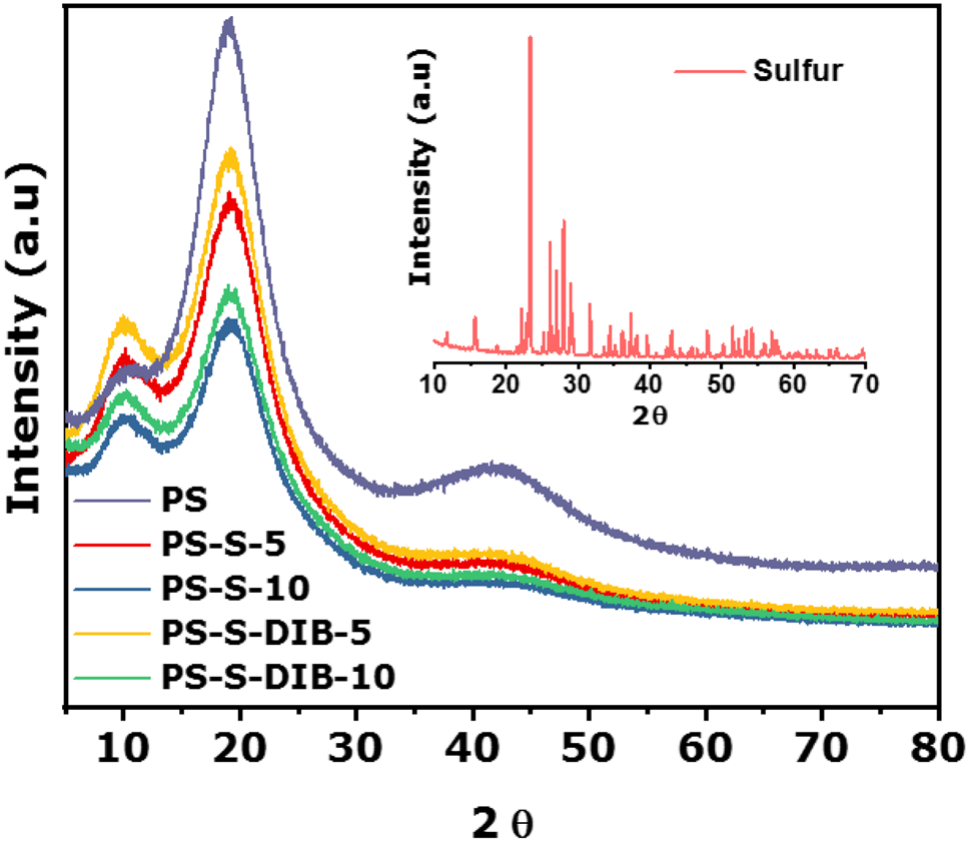
XRD of PS, PS-S, PS-SD composites and pure sulfur (insight graph).

Effect of the fillers on the structural behavior of polystyrene was analyzed by XRD. Figure 2 shows the XRD patterns comparison between PS with PS-S and PS-SD composites at different sulfur loading. PS shows a broad XRD peak at 2θ = 20° confirming its amorphous nature, similarly PS-S and PS-SD composites are also displayed amorphous peak in the same range, however; the peak intensities are reduced according to the sulfur content. It is also interesting to note that the sharp crystalline sulfur peaks (see insight image) are completely absent even in 10 wt% loaded composites samples, indicating the change in sulfur morphology after the extrusion. This can be explained as follows; during melt mixing process, both PS and polysulfur turns to viscous melts and hence the polysulfur chains can easily diffuse into the PS matrix under applied shear to form a homogeneous polymer blends. When these polymers are extruded they are allowed to cool naturally to the room temperature, during cooling process diffused polysulfur chains solidifies much faster than PS due to the difference in solidification rate. Thus, the mobility of polysulfur chains are arrested inside the PS matrix and results in random dispersion. Consequently, when these polysulfur chains revert back to the monomeric octasulfur form, their crystalline structure is destroyed which leads to the non-crystalline amorphous sulfur. In case of PS-SD the polysulfur chains reacts with the DIB to form highly cross-linked copolymers which further restricts the chain mobility and increases the amorphous nature. Thus, the sulfur crystalline peaks are completely absent in the composites. Furthermore, faster solidification of sulfur also interfere with the crystalline arrangement of PS chains and reduces the lamellar thickness, hence, increasing in sulfur loading resulted in decreased peak intensity. Similar behaviors was also observed in our previous work when sulfur is blended with high density polyethylene^40^.

Thermal stability of the PS-S and PS-SD nanocomposites was examined through thermogravimetric analyses (TGA). In Fig. S8, pure sulfur shows one step decomposition initiated around 200 °C and completely decomposes around 300 °C. However, nanocomposites showed two-step degradation, first step degradation started at around 205 °C was estimated to the decomposition of sulfur and is comparable to the amount of initial sulfur feed and the second decomposition step was by the polystyrene matrix. The amount of sulfur loading is comparable with the initial degradation of sulfur suggest complete incorporation of sulfur during extrusion processing.

**Figure 3:**
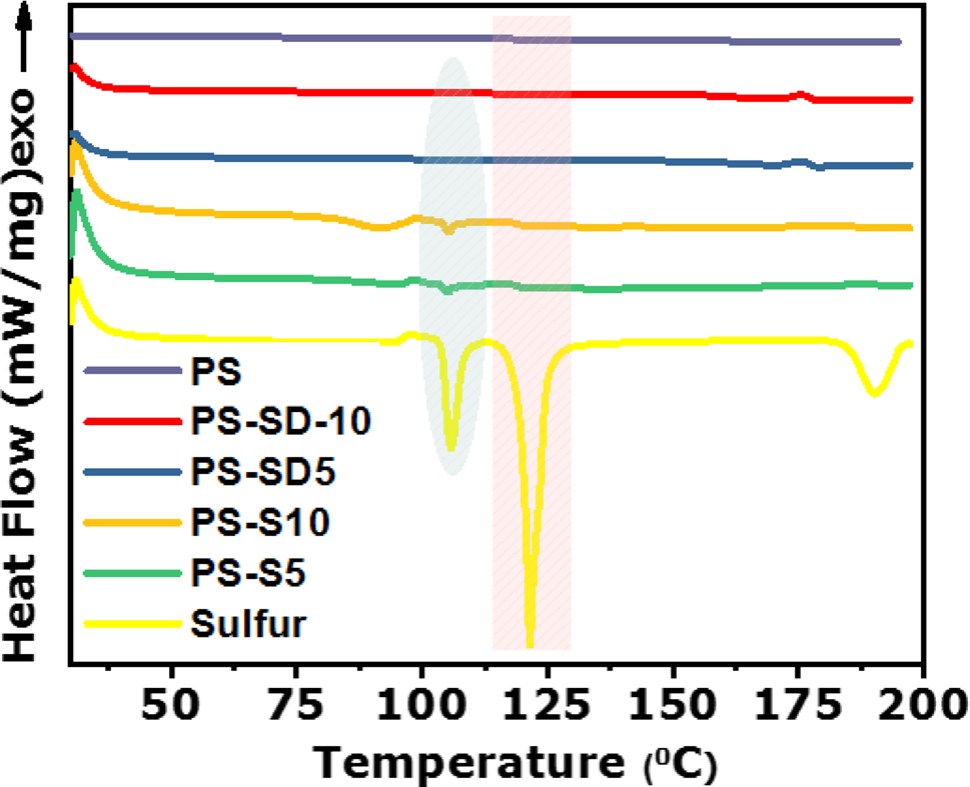
DSC thermogram of PS, PS-S and PS-SD composites.

In Fig. 3, DSC thermogram of pure sulfur shows three endothermic transitions at 106, 124, and at 184 °C, which are assigned for the solid–solid transition of sulfur from orthorhombic to monoclinic form, melting of monoclinic sulfur and for the polymerization of sulfur, respectively. However, these melt transition peaks are completely absent in both PS-S and PS-SD composites suggests that the crystalline sulfur changed to the amorphous form^41^. These results further supports the formation of amorphous sulfur and the absence of crystalline peaks in XRD.

**Figure 4:**
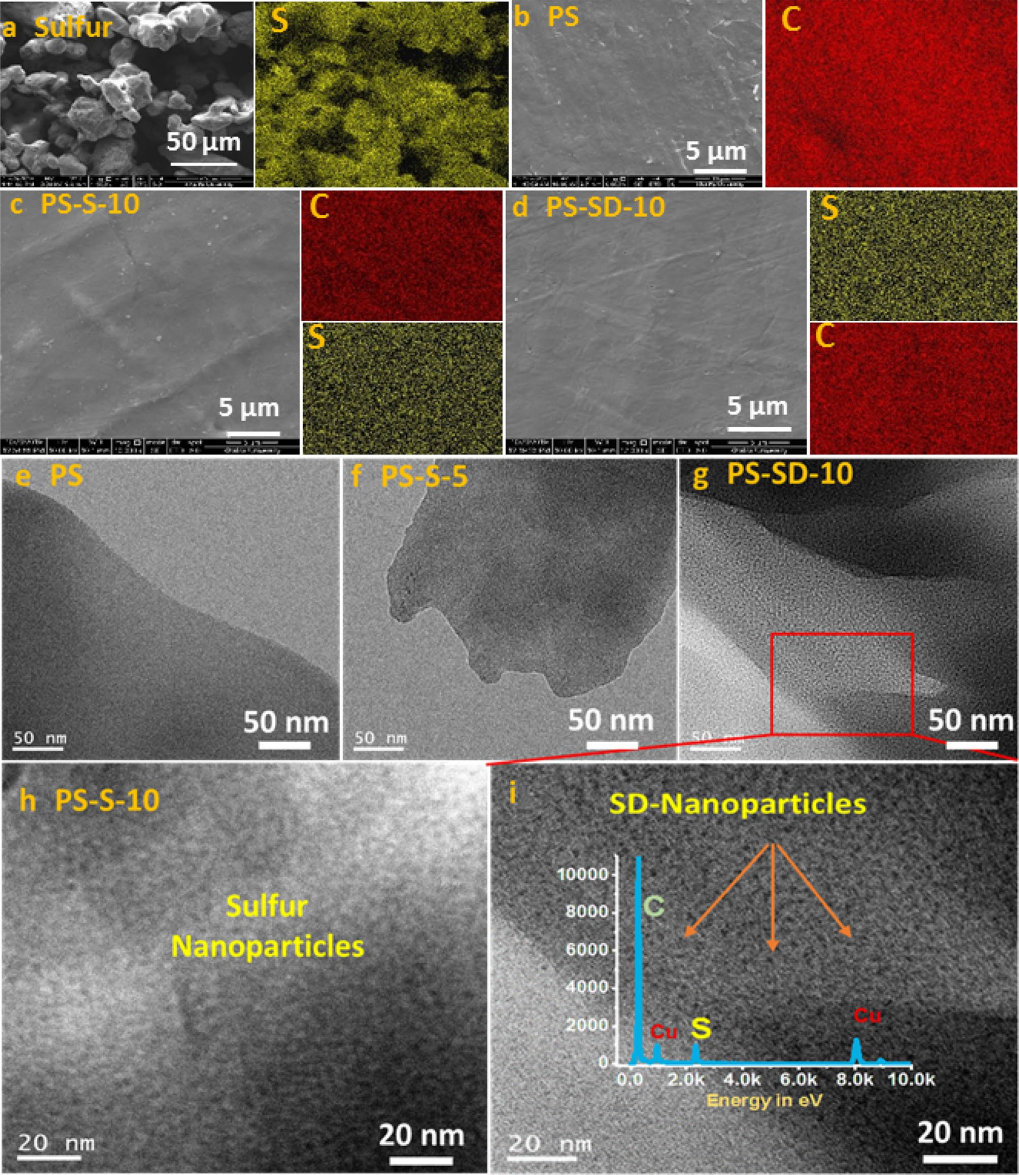
SEM images of (**a**) sulfur, (**b**) PS, (**c**) PS-S-10, (**d**) PS-SD-10 and their corresponding elemental imaging on the right side. TEM image of (**e**) PS, (**f**) PS-S-5, (**g**) PS-SD-10 and (**h**) PS-S-10. (**i**) Enlarged TEM image of PS-SD-10, insight graph show the EDX of the sample.

SEM and TEM images were used to study the surface morphology in the PS composites. Figure 4a shows the SEM image of elemental sulfur particles in random size, whereas neat PS in Fig. 4b display the uniform smooth surface, similar smooth surface was also observed in PS-S and PS-SD composites (se Fig. 4c,d) indicate high level of dispersion and compatibility of sulfur with PS matrix. EDX and elemental mapping of the composites confirms the presence of sulfur, however the particles are not observed in the SEM image due to their smaller size. Uniform distribution of sulfur and carbon elemental images further supports the formation of homogeneous composites. Sulfur dispersion and their particle size was studied by TEM using thin films prepared by ultramicrotome (films thickness around 100 nm). In Fig. 4e, PS shows plane surface, whereas, the samples PS-S-5 and PS-SD-10 displayed the uniformly dispersed dark particles on the smooth surface without any agglomeration (see Fig. 4f,g), these particles are completely absent in the pure polystyrene. Enlarged image of PS-S-10 and PS-SD-10 in Fig. 4h,i respectively, suggest the dark particles are around 5 nm in size and are arises due to the higher electron density of sulfur. EDX analyses further confirms the presence of sulfur in the dark spots. High transparence of the composites can be related to the nano size and non-agglomerated sulfur and SD particles.

**Figure 5:**
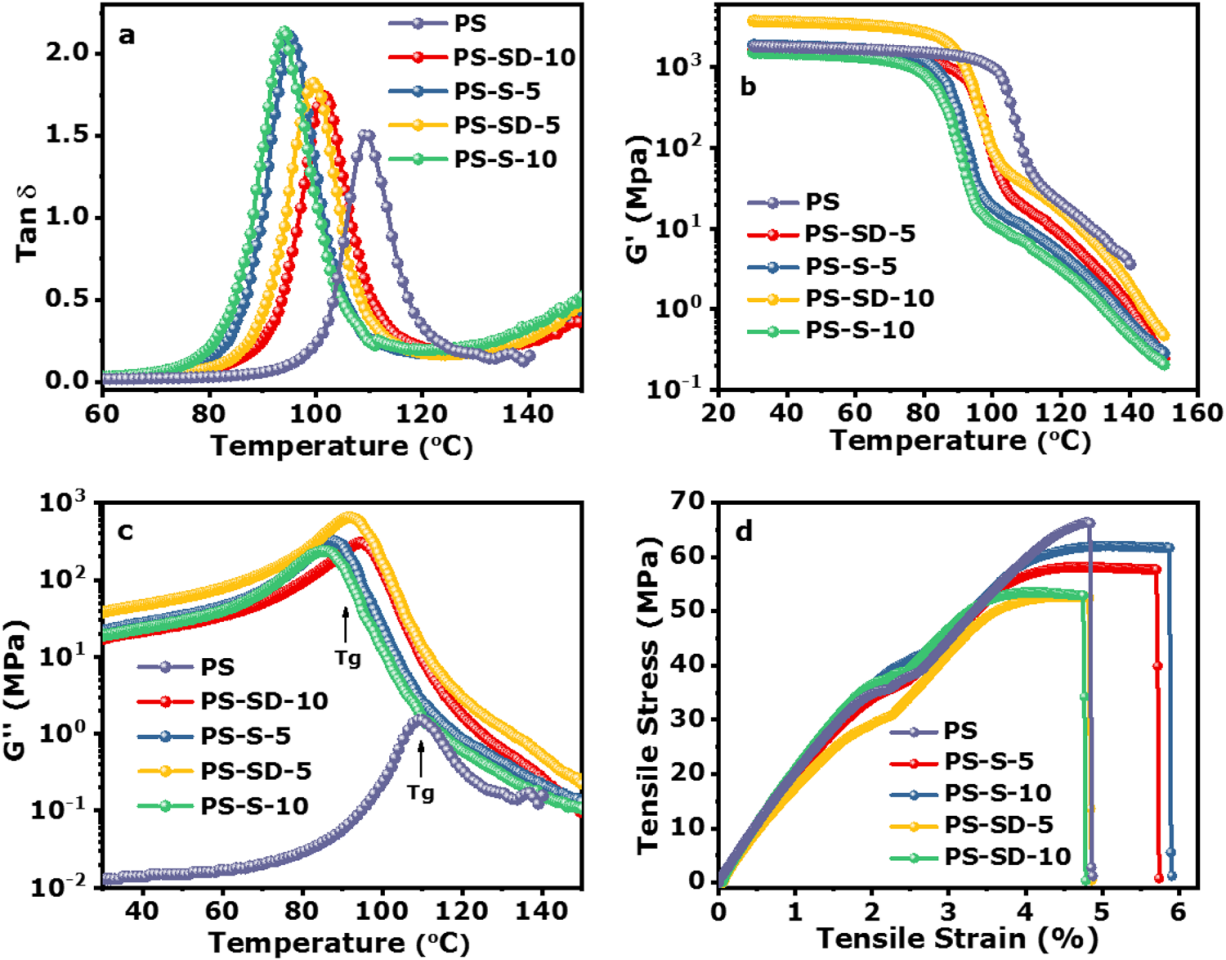
(**a**–**c**) Representative DMA curves, including tan δ, storage modulus (E′), and loss modulus (E″) of PS and its composites respectively. The experiments were carried out at a frequency of 1 Hz and a heating rate of 5 °C/min. (**d**) Tensile stress vs tensile strain curves of pure PS and their sulfur composites.

The mechanical properties of the PS and their composites were studied by DMTA and UTM. Figure 5a–c shows the loss tangent (tan δ), storage modulus (G′) and loss modulus (G″) versus temperature graphs of PS, PS-S-5, PS-S-10, PS-SD-5 and PS-SD-10, respectively. Single glass transition temperature (Tg) was observed in tanδ curve for PS, PS-S and PS-SD hybrid samples, shown in Fig. 5a–c and reported in Table 1. All the tanδ curves show uniformity with homogeneous nature of the hybrid materials. The tan δ curves of the PS, PS-S-5, PS-S-10, PS-SD-5 and PS-SD-10 hybrid materials show the glass transition temperature and tanδ max at 109.7 °C, 1.51; 95.4 °C, 2.01; 93.9 °C, 2.16; 99.3 °C, 1.84; and 102.2 °C, 1.76 , respectively. PS has a Tg of 109.7 °C, which was shifted to 95.4 °C and 93.9 °C after incorporation of sulfur (5% and 10%) into polymer matrix, suggest a unhindered segmental motion of PS chains. This could be due to the phase change of elemental sulfur, which act as a plasticizer in the hybrid materials. A comparison of glass transition temperature between PS-S and P-SD hybrid materials show that hybrid materials prepared from sulfur copolymer cross-linker (SD) were harder than the sulfur (S) based hybrid materials. This could be due to the strong cross-linking between the sulfur chains and formation of interpenetrating (IPN) network in PS matrix, which increased the cross-linked density in PS-SD-5 and PS-SD-10 hybrid materials. The observed Tg and storage modulus values for the series PS-SD-5 and PS-SD-10 show that, the Tg and crosslinking density increase with increasing SD content in the hybrid materials. Figure 5b,c shows the G′ and G′′ plots of PS, PS-S-5, PS-S-10, PS-SD-5 and PS-SD-10 hybrid materials. The storage modulus measures the stiffness and crosslinking density of the hybrid materials and loss modulus measures the glass transition temperature, reported in Table 1. Decrease in storage modulus was observed for the PS-S-5 and PS-S-10 hybrid materials compare to pure PS polymer and increase in storage modulus was observed for PS-SD-5 and PS-SD-10 compare to PS-S-5 and PS-S-10 hybrid materials at 40 °C and Tg + 5 °C region. However, storage modulus and crosslinking density both were decreased after incorporating sulfur and sulfur copolymer cross linker. The crosslink density (νe) of PS, PS-S-5, PS-S-10, PS-SD-5 and PS-SD-10 hybrid materials was calculated by using Eq. (1).

1 $$ \upsilon {\text{e }} = {\text{E}}^{\prime}\left( {{\text{G}}}^{\prime} \right)/{\text{3RT}} $$

where R is the universal gas constant, and T the temperature in K. G′ values in the rubbery region at T > Tg were taken to calculate υe by using Eq. (1).

**Table 1:** Glass transition temperature (Tg), storage modulus (E′) and crosslinking density (νe) data of PS-sulfur hybrid materials.

Sample name	Tg (E′′)	Tg (tanδ)	tanδ max	E′ (Pa) at (40 °C)	E′ (Pa) at (Tg + 5 °C)	νe, mol/cm^3^ E′ (40 °C)	νe, mol/cm^3^ E′ (Tg + 5 °C)
PS	109.2	109.7	1.51	1.85 × 10^9^	4.3 × 10^7^	2.36 × 10^–1^	4.44 × 10^–3^
PS-S-5	88.5	95.4	2.01	1.7 × 10^9^	1.8 × 10^7^	2.17 × 10^–1^	1.93 × 10^–3^
PS-S-10	85.6	93.9	2.16	1.44 × 10^9^	1.1 × 10^7^	1.84 × 10^–1^	1.19 × 10^–3^
PS-SD-5	91.5	99.3	1.84	1.8 × 10^9^	2.4 × 10^7^	2.3 × 10^–1^	2.55 × 10^–3^
PS-SD-10	95.6	102.2	1.76	3.8 × 10^9^	3.6 × 10^7^	4.86 × 10^–1^	3.8 × 10^–3^

The G′ values of PS-SD-5 and PS-SD-10 hybrid materials at temperatures 40 °C suggest the presence of good cross-linking and hardness. The glass transition temperature of the hybrid materials from tan δ curves and loss modulus (G′) curves shows very similar data, this could be due to the homogeneous arrangement of the molecular structure in the hybrid materials.

**Figure 6:**
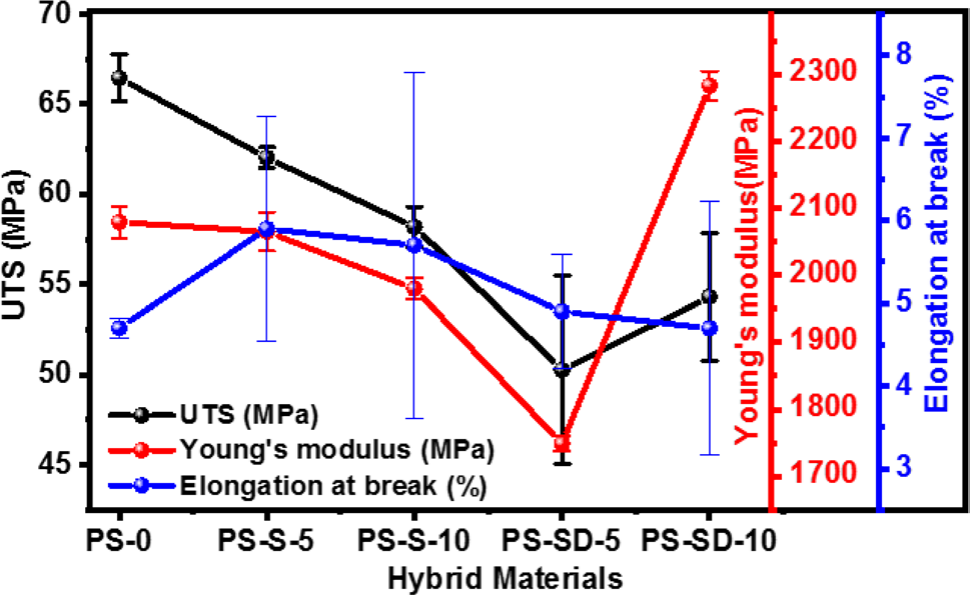
Effect of tensile strength, young’s modulus and elongation at break at different loading percentage of Sulfur and SD.

Effect of elemental sulfur and SD copolymer nanoparticles on tensile strength, elastic modulus and elongation at break of the PS-S and PS-SD hybrid materials were investigated. The stress vs strain curve and data are reported in Fig. 5d and Table 2. The UTS, Young’s Modulus and the elongation at break determined from Fig. 5d were presented in Fig. 6 show that UTS and Modulus decrease with increasing the elemental sulfur content. The ultimate tensile strength (UTS) of the PS-S and PS-SD hybrid materials slightly decreased for sulfur and SD loaded samples, i.e., 66.46, 62.03, 58.19, 50.27 and 54.31 MPa for PS, PS-S-5, PS-S-10, PS-SD-5 and PS-SD-10 hybrid samples respectively. However, with increasing SD concentration to 10 wt% resulted in increased UTS to 54.31 and modulus to 2283 MPa. This could be due to the formation of interpenetrating network (IPN) in the hybrid material. Interesting results were observed in elongation at break (%), where all the data close to same with slightly increase in elongation after addition of sulfur in PS polymer matrix. However, the mechanical properties of these PS-sulfur composites are much more superior to the inverse vulcanized copolymers (see Table 2)^42^. Inverse vulcanized materials are highly brittle to handle, whereas, in this case the PS-S and PS-SD composites exhibited mechanical strength similar to neat PS. The high mechanical strength and one step preparation method can be advantage to prepare in bulk by scale up process and can also be explored in wider application areas.

**Table 2:** Mechanical properties of PS-S and PS-SD hybrid materials compared with inverse vulcanized sulfur copolymers.

Sample name	Sulfur amount	Elongation (%)	UTS (MPa)	Young’s modulus (MPa)	References
PS	0	4.7 ± 0.12	66.46 ± 1.33	2079 ± 23.45	
PS-S-5	5	5.9 ± 1.36	62.03 ± 0.85	2065 ± 29.3	
PS-S-10	10	5.7 ± 2.10	58.19 ± 1.11	1980 ± 15.2	Present work
PS-SD-5	5	4.9 ± 0.69	50.27 ± 5.21	1749 ± 10.98	
PS-SD-10	10	4.7 ± 1.54	54.31 ± 3.54	2,283 ± 22.11	
N12	–	335 ± 10	52.9 ± 2.8	834 ± 7.2	Previous work
N12-S-2.5	2.5	270 ± 4.9	46 ± 2.2	929 ± 8.0	Ref.^42^
N12-S-5	5	260 ± 11.3	45 ± 0.9	945 ± 24	
N12-S-10	10	164 ± 10.2	41 ± 0.3	981 ± 4.6	
N12-S-20	20	139 ± 13.3	43 ± 1.1	895 ± 5.6	
N12-S-30	30	59 ± 17.3	37 ± 2.0	824 ± 21	
HDPE-Sulfur	30	932 ± 77	22.25 ± 2.7	638 ± 15	Ref.^40^
HDPE-Sulfur-	25	1,048 ± 56	22 ± 2.9	543 ± 40	
S-DAS	70	53.62	–	6.1	Ref.^41^
S-DIB	35	5.90 ± 0.52		146.23 ± 15	Ref.^43^
S-DIB	70	2.80 ± 1.80	17.5 ± 2.1	267.30 ± 51.49	
S-TIB	70	1.63 ± 0.51	19.5 ± 3.9	1,740 ± 150	Ref.^31^
S-TIB	50	2.33 ± 0.55	9.43 ± 3.06	1,100 ± 80	

Where; HDPE-High density polyethylene, DIB—1,3-diisopropenylbenzene, DAS-Diallyldisulfide, and TIB—1,3,5-Triisopropenylbenzene.

**Figure 7:**
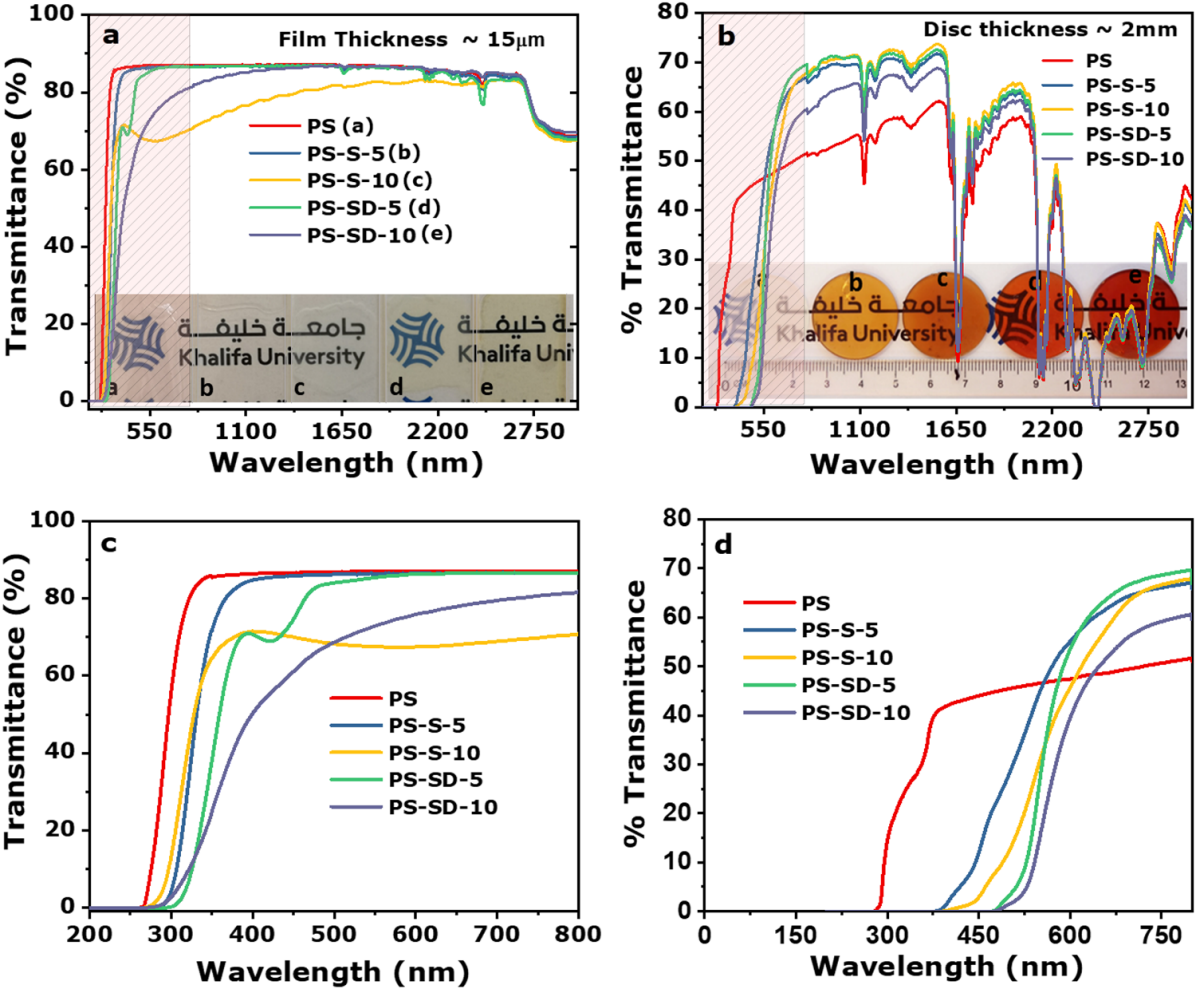
UV–Vis spectra of PS, PS-S and PS-SD thin films and molded samples.

Optical transparency of the PS composites was investigated using UV–Vis-near-IR transmition spectroscopy over a broad optical window (200–3,000 nm) to determine the effect of sulfur and samples thickness on optical properties. Samples with thickness around 15 μm and 2 mm were prepared by drop casting and injection molding method respectively. The thin films of PS, PS-S-5 and PS-S-10 appeared transparent, whereas, PS-SD-5 and PS-S-10 are slightly yellow in color. However, the molded samples of PS-S composites appear faint yellow and the inverse vulcanized PS-SD samples exhibits deep orange color (see insight image in Fig. 7a,b respectively). The thin films PS-S-5 and PS-SD-5 with 5 wt% sulfur loading are 85% transparent near IR region similar to pure polystyrene, however, increasing sulfur content to 10 wt% reduced the transparency to around 70%. Whereas, the optical transparency for thicker molded samples (2 mm disc) diminished to 60% in comparison to thin films (see Fig. 7a,b). This could be due to the increase in sample thickness as well as the absorbance of C-H vibrations from polystyrene and DIB co-monomer in the near-IR 1,500–3,000 nm range. Expended zone of the UV–Vis spectra between 200–800 nm for both thin films and molded samples are presented in Fig. 7c,d respectively. The spectra clearly indicate that the optical transparency of the PS-SD-10 thin films is higher than the PS-S-10. It has been observed that the larger nanoparticles aggregates in a polymer matrix steeply increase the intensity of scattered light, which would induce a dramatic decrease in the transparency^44^. Sulfur shows partial solubility in toluene, thus, the thin films prepared in toluene at lower sulfur concentration (5 wt%) showed higher transparency, however, when the loading increased the undissolved sulfur particles partially aggregates which resulted in reduced transparency. Whereas, in case of PS-SD samples the SD copolymers are completely soluble in toluene and hence form homogeneous solution of SD particles, thus displays higher transparency. In molded samples all samples displayed similar transparency around 60%. It is worth to note that the 15 µm‐thin films exhibit cutoff wavelengths (λcutoff) of 270–310 nm and the molded samples exhibited low transparency below 600 nm in the visible spectrum, as readily evidenced by the yellow to orange color of the composites material. However, the retention of high transparency in the visible (above 600 nm) was observed.

Sulfur containing polymer displayed HIR due to the high atomic refraction property of sulfur atom. Thus, the amount of sulfur and their degree of molecular packing in the polymers play an important role in controlling RI properties^1^. Hence, in recent studies, highly cross-linked sulfur rich copolymers prepared via inverse vulcanization using elemental sulfur displayed High Refractive Index (n > 1.7)^29^. In these sulfur rich copolymers the S–S bonds are largely IR inactive due to low organic content make them to use in IR imaging in near and mid-infrared region also and thus imparted high n to the sulfur copolymers and demonstrated the ability to use in IR imaging. The properties of these sulfur copolymers can be easily tunable by varying the cross-linkers amount.

**Figure 8:**
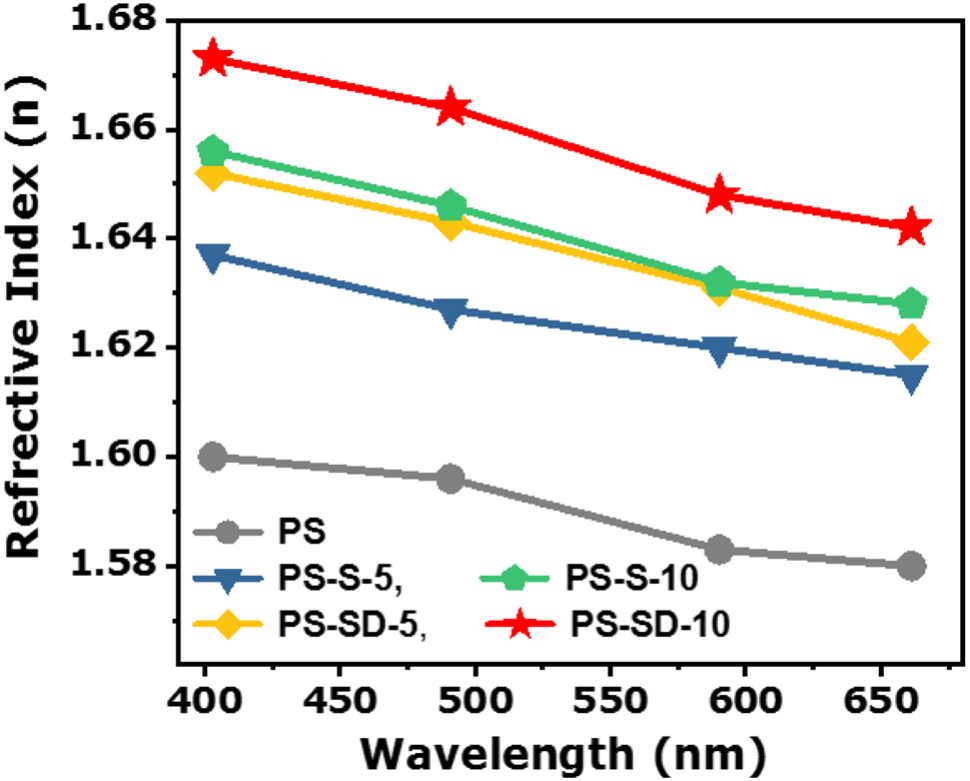
Comparison of Refractive Index of PS with PS-S and PS-SD composites.

Similarly, in current study the cross-linked sulfur nanoparticles with S–S bonds were generated inside the polystyrene matrix during extrusion process, these polymer composites can be easily molded in to different shape by molding or hot-press method. To measure RI, the composites were molded in to 2 mm thick circular molds and the variation in RI with respect to the wavelength are measured and are presented in Fig. 8. Pristine PS shows RI n = 1.601 at 402.9 nm, whereas, addition of elemental sulfur in the matrix increased the RI n = 1.656, and RI increased with increase in the sulfur loading confirms the role of S–S bonds in improving RI. Cross-linking these sulfur with DIB further improved the RI to 1.652 to 1.673 according to the SD copolymers loading (5 and 10 wt%) respectively. The higher RI of PS-SD compared to the PS-S are due to the higher dipolar arising from the cross-linked structure and the presence of aromatic DIB in PS matrix.

Abbe’s number (ν_D_), a key parameter to measure refractive index dispersion, is also an important for optical materials used in the visible region. ν_D_ can be calculated from bellow Eq. (2)

2 $$ \nu_{{{\text{D}} }} = n_{D} - { 1}/n_{F} - n_{C} $$

where: n_D_, n_F_, and n_C_ are the refractive indices of the material at the wavelengths of the sodium D (589.3 nm), hydrogen F (486.1 nm), and hydrogen C (656.3 nm) lines, respectively.

The Abbe’s numbers calculated from Eq. (2) for pristine PS, PS-S and PS-SD at 486, 589, and 656 nm wavelength using RI in Fig. 8 are calculated from Table S1. The obtained high Abbe’s number for the PS-S and PS-SD molds are in the range ~ 30 ± 1, indicate the high level dispersion of sulfur and SD copolymer in PS matrix, this also supports the increased RI in the composites.

## Conclusion

In summary, this work demonstrate the simple, scalable method to achieve high RI material by direct utilization of elemental sulfur via in situ inverse vulcanization and melt extrusion technique. The prepared PS-S and PS-SD composites are transparent and showed high transparency depending on the film thickness. Further, TEM images confirmed the homogeneous dispersion of both sulfur and SD nanoparticles in the PS matrix without any visible aggregates. Absence of sulfur crystalline peaks in DSC thermogram supported the conversion of crystalline sulfur into uniformly dispersed amorphous form in the PS matrix. DMTA study indicate the slight reduce in the Tg with the increasing filler loading due to the plastization effect of sulfur under heat treatment. Mechanical properties of the composites are comparable to the pristin PS, hence can be used in wide application without sacrificing the strength. RI of PS n = 1.601 at 402.9 nm was increased to n = 1.656 and 1.673 by the incorporation of 10 wt% of sulfur and SD copolymer nanoparticles respectively in the composites. Higher RI of PS-SD then PS-S composites are due to the uniform dispersion and improved dipolar arise from the cross-linked structure of sulfur and DIB. The main advantage of the method is that the RI can be controlled by the addition of sulfur and DIB during extrusion process and the obtained materials can be easily processed in to different shapes by adopting both melt and solution processing techniques, moreover the proposed method can also be extended to the other melt processable polymers.

## Materials and methods

### Materials

Polystyrene pellets, 5 mm was obtained from sigma-Aldrich chemicals (USA), Powdered Sulfur (> 99% purity) supplied by Merck chemical (Germany), and 1,3-diisopropenylbenzene (DIB, 97%) supplied by (TCI America) were used without further purification.

### Preparation of polystyrene composites

Polystyrene-sulfur (PS-S) and polystyrene-S/DIB (PS-SD) nanocomposites were prepared by one step melt extrusion method using twin-screw Haake Minilab II extruder. The extruder temperature for all preparation was maintained at 230 °C with an average screw rotation of 100 rpm and the mixing time of 30 min. The extruder was kept inside fume wood and the extrusion were carried out under N_2_ atmosphere in presence of a H_2_S gas detector for all experiments as percussion. The PS-SD nanocomposites were prepared by the in situ inverse vulcanization of S-DIB in polystyrene matrix under extrusion conditions by keeping cross-linker (DIB) amount at 30 weight percent with respect to the sulfur content. A representative example for the preparation of PS-SD-5 containing 5 weight % sulfur is as follows. Polystyrene (4.75 g) is melted inside the extruder at 230 °C, to this 0.25 g sulfur (5% with respect to PS) and 0.075 g DIB (30 wt% with respect to sulfur) was added and allowed to mix for 30 min. The composite was then extruded and allowed to cool down to room temperature naturally. The samples were termed as PS-SD-X composites (X = sulfur content, 5 or 10 wt%). A similar conditions was applied to prepare PS-S composites except by adding DIB cross-linker. The composites were denoted as PS-S-Y (Y = sulfur content, 5 or 10 wt%). Pure polystyrene was also extruded which serves as reference. The extruded materials were then chopped into small pieces and molded in to desired shapes by injection molding. Injection molding was carried out by using Thermo Scientific Haake minijet pro injection molder at cylinder temperature 190 °C and mold temperature 115 °C and the injection pressure kept at 500 bar. All the molded samples have uniform thickness of 2 mm. Thin film were prepared by using hot-press just above the melt temperature of the polystyrene.

### Characterization

Melt extrusion of the polymer was carried out using twin-screw Haake Minilab II extruder with screw diameter 5/14 mm conical and screw length 109.5 mm. Infrared spectra of the PS, PS-S and PS-SD composites were recorded by Attenuated Totally Reflectance Fourier Transformed Infrared spectroscopy technique (FTIR-ATR), using a Bruker Vertex 70. Diffraction (XRD) patterns were collected using analytical X’Pert PRO Powder Diffractometer (Cu-Ka radiation 1.5406 Å, 40 kV, 40 mA) in the range of 5°–80° 2θ scale, with a step size of 0.02°. The thermal degradation behavior was studied in TGA Netzsch Sta. 409 PC/PG (Germany). The samples (10–15 mg) were scanned from 25 to 600 °C at a heating rate of 10 °C/min in nitrogen environment. Differential Scanning Calorimetry (DSC) analysis of the composite samples (5–10 mg) was done using DSC Netzsch (Germany) at a heating rate of 10 °C/min in the temperature range between − 0 and 200 °C in a nitrogen environment. The dynamic mechanical behavior of the samples was studied using dynamic mechanical analyzer (TA Instruments, DMAQ800). The experiments were carried out a fixed frequency of 1 Hz and at a heating rate of 2 °C/min. The tests were conducted in a temperature range of 25 to 150 °C using rectangular samples of dimensions with (25 mm × 12 mm × 0.5 mm). (TEM) images were obtained using FEI Tecnai G20 operated at 200 kV accelerating voltage to observe the nanoscale structures of the sulfur in the composite. Samples were ultra-microtomed at room temperature conditions to prepare less than 100 nm thick samples. Refractive index of the samples were determined by using nulling imaging ellipsometer (Nanofilm_ep3se + NIR upgrade, Accurion). The incident elliptically polarized light was reflected off the sample onto a detector through an objective and a polarizer. The ellipsometric null condition was fulfilled when the incident light ellipticity, was selected so that the reflected light was completely linearly polarized and an analyzer (A) before the detector was in such position that the absolute minimum of light flux was detected. The shape of the incident light was controlled by positions of a polarizer (P) and a lambda quarter-wave plate (C). The positions of P, C, A at the nulling condition are related to the optical properties of the sample. Reduction of the measured data with computerized optical modeling leads to a deduction of complex refractive index. Initially, an angular dependence at selected wavelengths were measured for each sample. The obtain data was used to determine complex index or refraction for each selected wavelength, using supplied modeling software (EP4-model, Accurion). In modeling software, surface roughness and inclusion of voids during pouring were take into account.

## Supplementary information

Supplementary Information
