# Supplementary material for: Scalable High Refractive Index polystyrene-sulfur nanocomposites via in situ inverse vulcanization
Source: Sci Rep. 2020 Sep 10;10:14924. doi: 10.1038/s41598-020-71227-z (PMC7483506; doi:10.1038/s41598-020-71227-z)
Supplement: Supplementary file 1 — Supplementary Information [file 41598_2020_71227_MOESM1_ESM.docx]

Supplementary Information

**Scalable High Refractive Index Polystyrene-Sulfur Nanocomposites via In Situ Inverse Vulcanization**

Vijay S Wadi ^a^*, Kishore K. Jena ^a^, Kevin Halique ^a^, Brigita Rožič ^b^, Luka Cmok ^b^, Vasileios Tzitzios ^c^ and Saeed M. Alhassan ^a^*

^a^ Department of Chemical Engineering, Khalifa University of Science and Technology,

PO Box 127788, Abu Dhabi, United Arab Emirates

^b^ Institut “Jožef Stefan”, P.O. Box 3000, 1001 Ljubljana, Slovenia

^c^ NCSR "Demokritos" Institute of Nanoscience and Nanotechnology, Athens

15310, Greece

*Corresponding Author:

Saeed M. Alhassan, e-mail: [saeed.alkhazraji@ku.ac.ae](mailto:saeed.alkhazraji@ku.ac.ae)

Vijay S Wadi, e-mail: vijay.wadi@ku.ac.ae


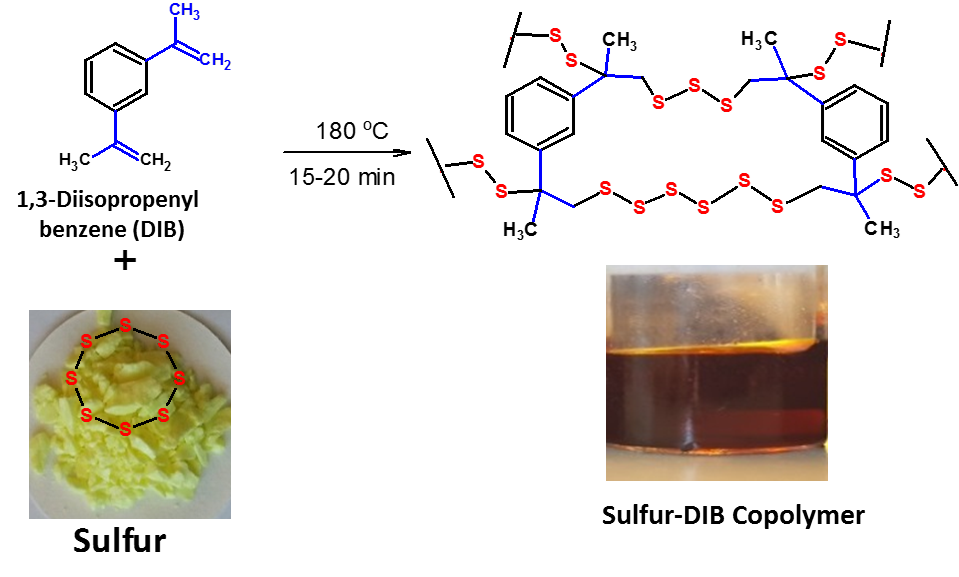


**Figure S1** In situ inverse vulcanization of sulfur and DIB at 185 ^o^C, yellow color of sulfur changed to orange red after the cross-linking reaction, similar color change was also observed during in situ inverse vulcanization reaction.


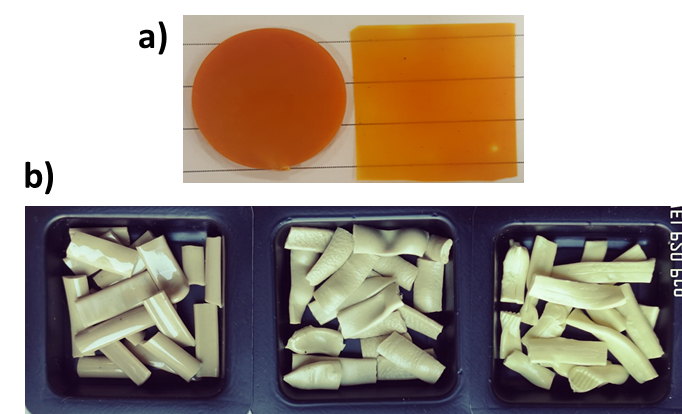


**Figure S2** a) PS-SD sample with 15 wt% sulfur, thick molded sample (left) completely opaque whereas, hot-pressed samples shows reduced transparency. Reduced in the transparency could be due to the excess of sulfur agglomerated during extrusion and molding process.

b) From left to right- extrudate of PS-S-15, PS-S-20 and PS-S-25, with 15, 20 and 25 wt% of sulfur loading respectively. The PS-S sample above 10 wt% of sulfur became opaque and non-transparent similar to the PS-SD samples and above 20 wt% sulfur start to leach out from the matrix. The increased intensity of yellow color sulfur and rough powdery appearance on the surface of PS-S-25 indicate the leaching of sulfur from the PS matrix.


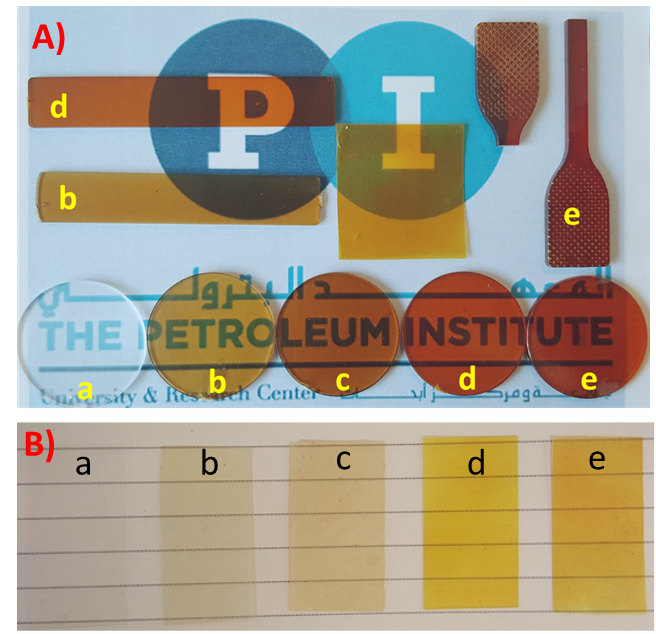


**Figure S3** (A) Injection molded polystyrene composite samples in different shape and size used for mechanical and thermal testing, samples showing good transparency at 2 mm thickness (B) hot-pressed thin films prepared using extrudates. Where: a) pure PS, b) PS-S-5, c) PS-S-10, d) PS-S-DIB-5 and e) PS-S-DIB-10 respectively


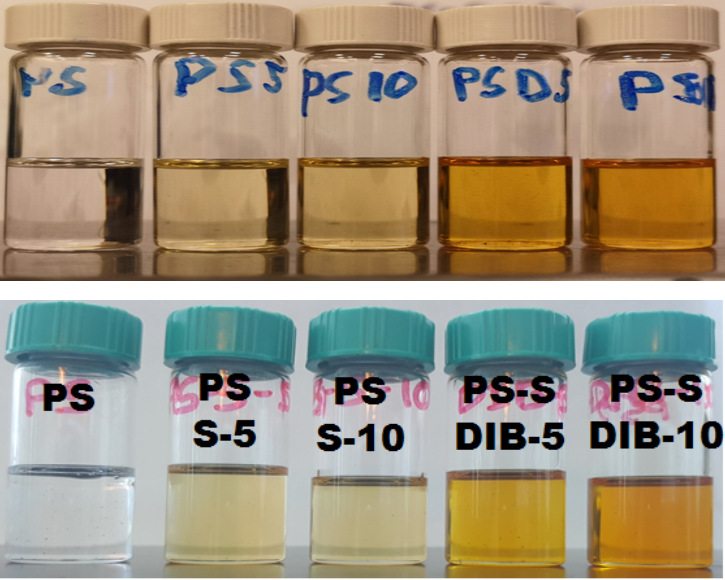


**Figure S4** Dissolved pure polystyrene and PS-S and PS-SD composites in (top) toluene and bottom in DMF


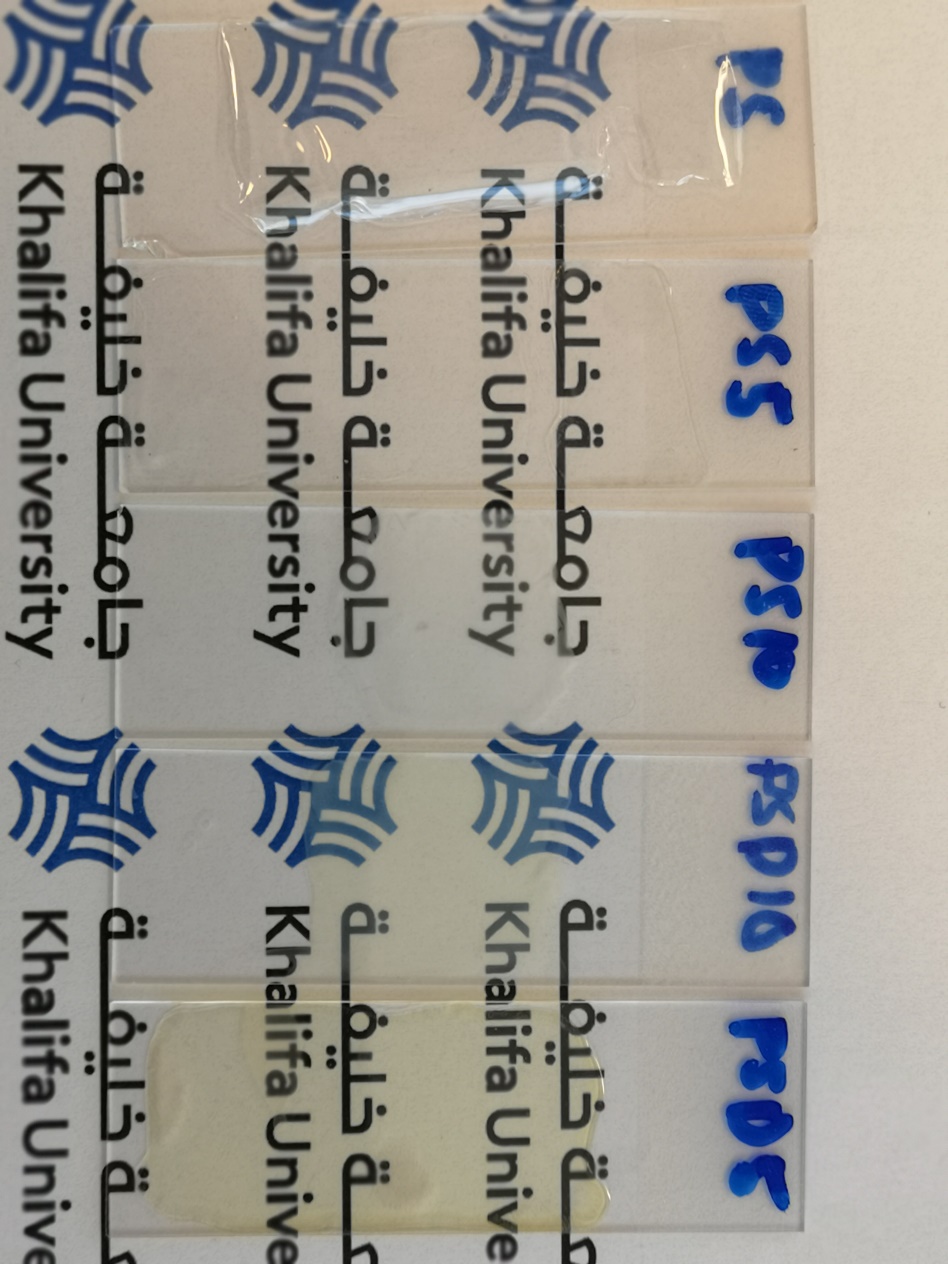


**Figure S5** Thin films prepared by solution casting method in toluene using 5wt% samples

**Figure S6** FTIR spectrum of PS and its sulfur composites


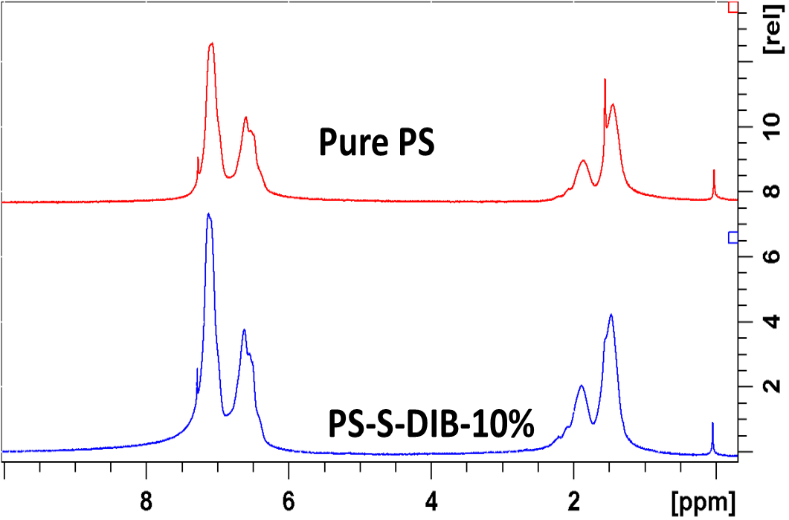


**Figure S7** Solid state ^1^H-NMR spectra of Pure PS and PS-SD-10 composites, the peaks of polystyrene are unchanged after processing indicate the stability of PS against sulfur under extrusion condition

**
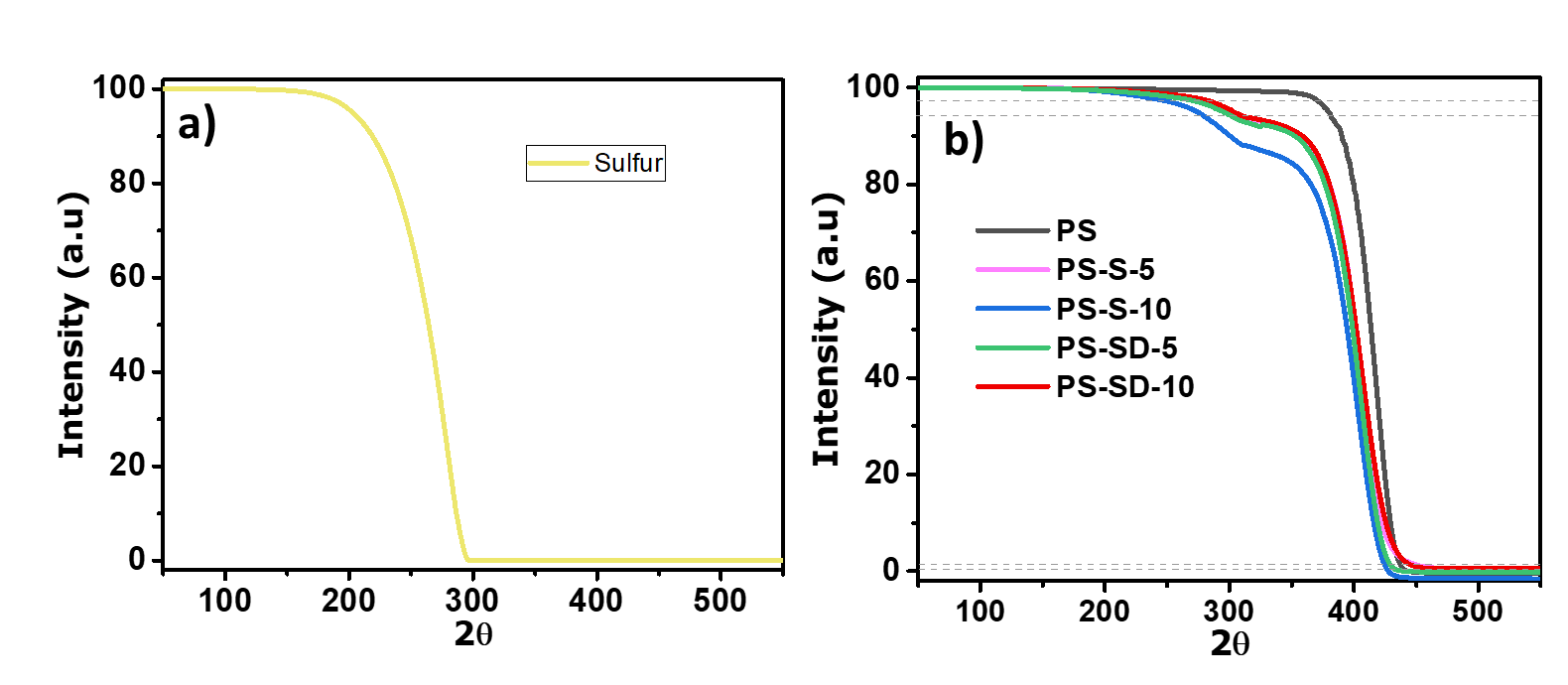
**

**Figure S8** TGA Thermogram of a) pure sulfur and b) Polystyrene-sulfur composites. Sulfur degradation initiated around 205 ^o^C in the composites and completely decomposed around 300 ^o^C similar to pure sulfur and the weight loss is proportional to the total weight of sulfur loaded in the composites.

**Table S1** Optical Properties of the Polystyrene-Sulfur composites

| **Samples** | **Sulfur wt % ^a^** | **DIB wt % ^b^** | ***n_486.1_*** | ***n_589.1_*** | ***n_656.3_*** | **ν_D_ ^c^** |
| --- | --- | --- | --- | --- | --- | --- |
| PS | -- | -- | 1.601 | 1.591 | 1.582 | 31.10 |
| PS-5 | 5 | -- | 1.628 | 1.624 | 1.602 | 29.71 |
| PS-10 | 10 | -- | 1.641 | 1.631 | 1.622 | 30.50 |
| PS-SD-5 | 5 | 1.5 | 1.646 | 16.32 | 1.625 | 29.40 |
| PS-SD-10 | 10 | 3 | 1.658 | 1.648 | 1.636 | 29.45 |

^a^ Sulfur weight % with respect to total weight of polystyrene, ^b^ DIB weight 30% with respect to sulfur, ^c^ Abbe’s number (ν_D_) is calculated by ν_D_ = *n*_589.3_ - 1/*n*_486.1_ - *n*_656.3_.
